# Sodium‐enriched nectar shapes plant–pollinator interactions in a subalpine meadow

**DOI:** 10.1002/ece3.70026

**Published:** 2024-07-16

**Authors:** Ethan VanValkenburg, Thiago Gonçalves Souza, Nathan J. Sanders, Paul CaraDonna

**Affiliations:** ^1^ Department of Ecology and Evolutionary Biology University of Michigan Ann Arbor Michigan USA; ^2^ Rocky Mountain Biological Laboratory Gothic Colorado USA; ^3^ Institute for Global Change Biology University of Michigan Ann Arbor Michigan USA; ^4^ Chicago Botanic Garden Glencoe Illinois USA; ^5^ Program in Plant Biology and Conservation Northwestern University Evanston Illinois USA

**Keywords:** *Bombus* spp., community ecology, micronutrients, networks, plant–pollinator interactions, sodium

## Abstract

Many plants have evolved nutrient rewards to attract pollinators to flowers, but most research has focused on the sugar content of floral nectar resources. Concentrations of sodium in floral nectar (a micronutrient in low concentrations in nectar) can vary substantially both among and within co‐occurring species. It is hypothesized that sodium concentrations in floral nectar might play an important and underappreciated role in plant–pollinator interactions, especially because many animals, including pollinators, are sodium limited in nature. Yet, the consequences of variation in sodium concentrations in floral nectar remain largely unexplored. Here, we investigate whether enriching floral nectar with sodium influences the composition, diversity, and frequency of plant–pollinator interactions. We experimentally enriched sodium concentrations in four plant species in a subalpine meadow in Colorado, USA. We found that flowers with sodium‐enriched nectar received more visits from a greater diversity of pollinators throughout the season. Different pollinator species foraged more frequently on flowers enriched with sodium and showed evidence of other changes to foraging behavior, including greater dietary evenness. These findings are consistent with the “salty nectar hypothesis,” providing evidence for the importance of sodium limitation in pollinators and suggesting that even small nectar constituents can shape plant–pollinator interactions.

## Graphical abstract

Sodium concentrations in floral nectar might play an important and underappreciated role in plant–pollinator interactions. In a field experiment, we find that flowers with sodium‐enriched nectar received more than twice as many visits from a greater diversity of pollinator visitors throughout the season with concurrent shifts in dietary breadth and community composition. These results highlight the importance of sodium limitation in plant–pollinator communities and suggest that even small nectar constituents can significantly shape interactions.

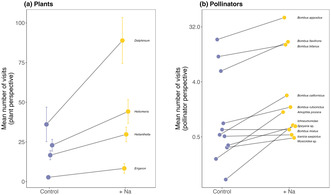

## 1 INTRODUCTION

Nutrient availability determines the structure, diversity, and function of ecological communities (Hunter & Price, 1992). While nitrogen, phosphorus, and potassium have received considerable attention (Burkle & Irwin, 2009; Sterner & Elser, 2002), micronutrients are now receiving increased appreciation for the myriad processes that they mediate in ecological communities (Kaspari & Powers, 2016). Sodium (Na), in particular, has been the focus of a growing number of studies (Kaspari, 2020) because it plays a key role in maintaining metabolism, fluid balance, and costly Na‐K ATPases in animals (Kaspari, 2020). Growing evidence suggests that herbivores respond to sodium availability because they need sodium to function, but plants are not typically rich in sodium (Prather et al., 2018; Welti et al., 2019). Therefore, many herbivores must exploit sodium‐rich resources (Kaspari, 2020). When sodium occurs at high concentrations, either naturally (e.g., as mineral deposits or in bovine urine) or experimentally, the activity of herbivores increases. For example, sodium found in saltlicks or salt deposits attract cattle (Hebert & Cowan, 2011; Kennedy et al., 1995), sodium‐enriched food resources attract foraging ants (Kaspari et al., 2008, 2020), and sodium additions in a prairie increase the diversity and abundance of insects (Prather et al., 2018).

Pollinators depend extensively on flowers to meet their nutritional demands (Michener, 2000; Willmer, 2011), but there is also a dearth of sodium in floral nectar (Filipiak et al., 2023; Hiebert & Calder, 1983) and pollen (Filipiak et al., 2017). As a result, some pollinators have been observed to exploit non‐floral sodium‐rich resources: human sweat and tears attract sweat bees (Bänziger et al., 2009), stingless bees supplement electrolytes from carrion (Dorian & Bonoan, 2021), and butterflies exhibit puddling behaviors in salty water (Arms et al., 1974). Pollinators may also exploit the substantial variation in sodium concentration in nectar within and among co‐occurring plant species (Hiebert & Calder, 1983). A recent field experiment demonstrated that spiking floral nectar with sodium consistently led to a greater number of flower visits from a more diverse set of pollinator visitors across five different plant species (Finkelstein et al., 2022a). For plants, the redundancy from diversity may improve pollination services (Loy & Brosi, 2022) and buffer against the loss of individual pollinator species (Winfree & Kremen, 2009). But, sodium in floral nectar might do more than simply attract more pollinators. For example, other measures of floral nectar rewards (volume, sugar concentration, and resource diversity) can strongly influence the community composition of floral visitors (Potts et al., 2003). For pollinators, sodium‐enriched nectar might shape the frequency of their floral visits, breadth of interactions, and dietary composition. Not all pollinator species respond similarly to changes in nectar resources (Barberis et al., 2023; Willmer, 2011), so their response to sodium‐enriched nectar resources may differ between pollinator species.

Here, we test the effect of experimentally sodium‐enriched nectar on pollinator visitation and diversity in an intact subalpine meadow ecosystem using wild plants and pollinators in the Colorado Rocky Mountains, USA. We expand on previous work (Finkelstein et al., 2022a) by examining the behavioral responses of different species of floral visitors (e.g., visitation rate and handling time), their diet breadth, and the community composition of floral visitors to plants. Our experiment uses nectar volumes and sugar concentrations that approximate naturally occurring floral nectar in our study system. Using this experimental setup, we ask four interrelated questions about sodium‐enriched floral nectar and plant–pollinator interactions: (1) Do plants with greater sodium nectar concentrations attract more floral visits by more species? (2) Do pollinator species preferentially visit flowers with greater concentrations of sodium in their nectar? (3) Does sodium‐enriched floral nectar significantly alter the community composition of floral visitors? (4) Do pollinators change their diet breadth in response to increased sodium concentrations in floral nectar? We hypothesize that if pollinators are sodium‐limited, they will preferentially forage on sodium‐rich resources, leading to a greater frequency and diversity of pollinator visitors on plants with sodium‐enriched nectar.

## 2 METHODS

### 2.1 Site description

We conducted floral nectar sodium addition experiments in a subalpine meadow near the Rocky Mountain Biological Laboratory (RMBL) in Gothic, Colorado, USA (Marriage Meadow, 38°58′ N, 106°59′ W, 2900 m above sea level) between July 8 and August 2, 2022. The study area is a mosaic of wet and dry meadows, interspersed with aspen and conifer forests. This subalpine ecosystem is exemplified by a diverse community of native plants and pollinators, and notably the absence of *Apis mellifera* (Eurasian honeybee) which can alter the behavior of native foraging pollinators (CaraDonna et al., 2017; Ogilvie & CaraDonna, 2022). Common plant species flowering at the site during our experiment include *Aquilegia coerulea*, *Delphinium barbeyi*, *Erigeron speciosus*, *Helianthella quinquenervis*, *Heliomeris multiflora*, and *Ipomopsis aggregata*.

### 2.2 Experimental design

**FIGURE 1:**
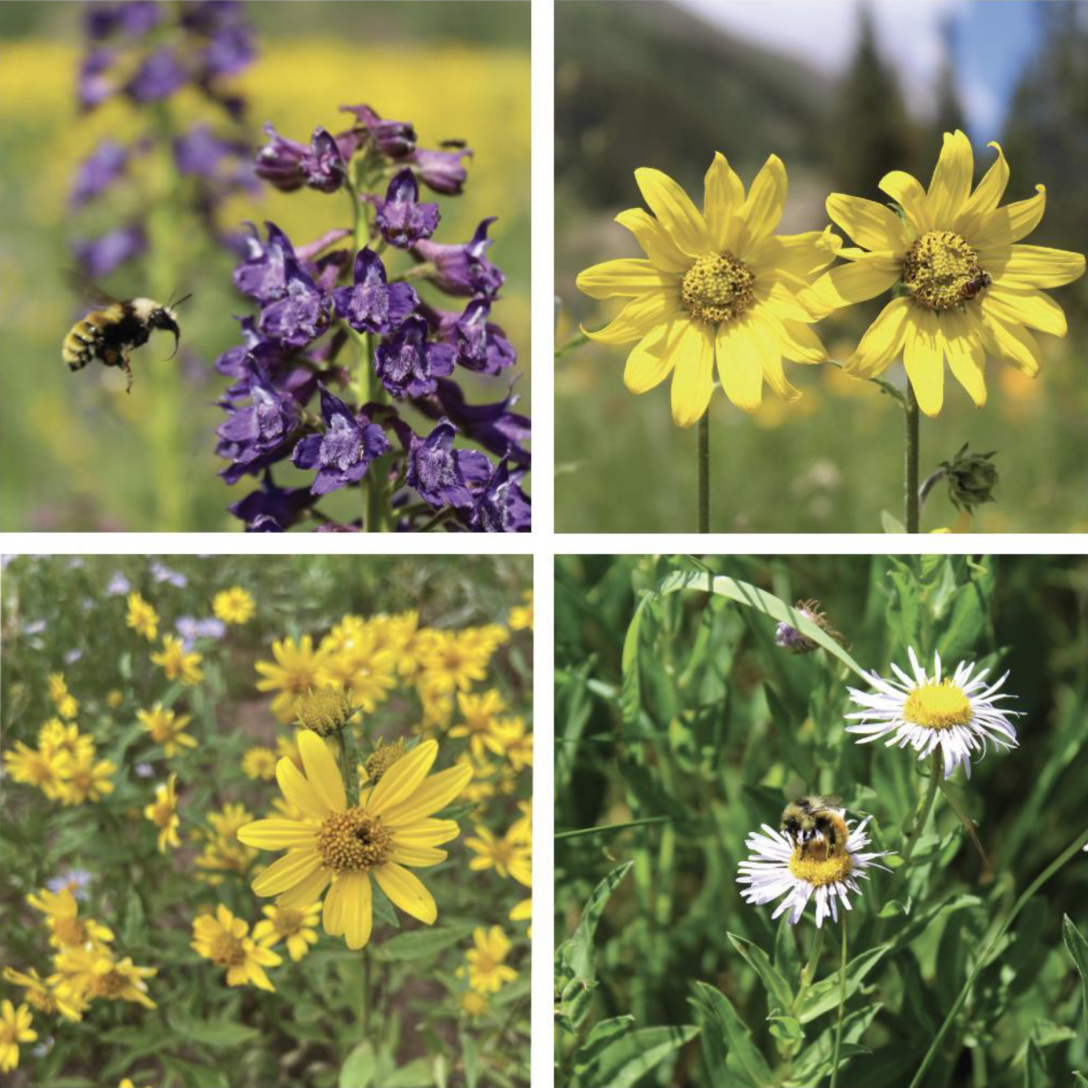
Focal plant species included in the experiment. Clockwise from top left: *Delphinium barbeyi*, *Helianthella quinquenervis*, *Erigeron speciosus*, and *Heliomeris multiflora*. We define a plant–pollinator interaction when a floral visitor comes into contact with the reproductive structures of the flower (see visits on *H. quinquenervis* and *E. speciosus*). In many cases, extended tongues, like that of *Bombus appositus* approaching *D. barbeyi*, indicate pollinators were foraging for nectar.

We selected four focal species for this experiment that co‐occur, are abundant, and co‐flower (in July) within our study site (Figure 1): *Delphinium barbeyi* (Ranunculaceae), *Erigeron speciosus* (Asteraceae), *Helianthella quinquenervis* (Asteraceae), and *Heliomeris multiflora* (Asteraceae). Additionally, the selected plant species have abundant and diverse communities of floral visitors and differ in their floral traits (Bain et al., 2022; CaraDonna & Waser, 2020). For each focal species, we selected five paired plants to use for the duration of the experiment. Plant pairs were typically within 1 m of one another, similarly sized, and had a similar number of flowers. For *D. barbeyi* and *E. speciosus*, we selected individual plants for replicate pairs, and for *Helianthella quinquenervis* and *Heliomeris multiflora*, we considered adjacent groupings (2–5 individuals clustered together) of plants as a single replicate because each individual plant has few or only one inflorescence. Within each pair of plants for all species, the flowers of one plant (or cluster of plants) received artificial control nectar additions and the other received artificial sodium‐enriched nectar additions. Nectar treatment assignments within each replicate pair were the same for the duration of the experiment. Control nectar contained 50% sucrose (weight: volume) and sodium‐enriched experimental nectar contained 50% sucrose +0.5% NaCl (weight: volume). To make the artificial nectar solutions, we combined 170 g of sugar and 200 mL of water for 0.5°Bx. For the sodium‐enriched treatment, we added 1.55 g NaCl (~0.5% NaCl w:v). The amount of artificial nectar applied to the flowers approximates naturally occurring nectar volumes and sugar concentrations observed at the Rocky Mountain Biological Laboratory (Table 1; Hiebert & Calder, 1983; Luo et al., 2014; Kirschke and CaraDonna, unpublished data). Observations of sodium concentrations in floral nectar of 14 species over 2 years in the Colorado Rocky Mountains range from <0.001% Na to 0.12% Na (Hiebert & Calder, 1983). Therefore, the 0.5% NaCl (0.077% Na) sodium‐enriched nectar was approximately 5× naturally occurring concentrations of sodium in *D. barbeyi* at the RMBL (Table 1). We push the sodium concentration beyond its natural conditions to test the effect of sodium in nectar, recognizing that naturally occurring nectar may dilute our treatments.

**TABLE 1:** Observed naturally occurring and experimental nectar traits for focal species included in this experiment. Volume and sugar concentrations from G. Kirschke and P. CaraDonna. Ambient sodium concentrations from Hiebert and Calder (1983).

Species	Observed			Experimental treatment			
	Volume (μL) mean ± SD [max]	Sugar (mg/μL) mean ± SD	Na% (w:v)	Volume (μ)	Sugar (mg/μL)	NaCl % (w:v)	Na% (w:v)
*Delphinium barbeyi*	1.30 ± 1.08 [5.37]	0.67 ± 0.26	0.006 ± 0.001	2.5	0.50	0.5	0.07
*Erigeron speciosus*	0.20 ± 0.13 [0.53]	0.77 ± 0.18	–	1.2	0.50	0.5	0.07
*Helianthella quinquenervis*	0.42 ± 0.34 [1.63]	0.72 ± 0.26	–	1.5	0.50	0.5	0.07
*Heliomeris multiflora*	1.54 ± 0.65 [3.25]	0.68 ± 0.18	–	1.5	0.50	0.5	0.07

### 2.3 Sodium enrichment of nectar and flower visitor observations

We applied the experimental nectar treatments and observed interactions between flowering plants and insect pollinators on warm, sunny days. Nectar treatments were randomly assigned to each of the plants in every pair and applied by gently inserting a pipette tip into the nectar spur (*D. barbeyi*), into individual florets (*H. quinquenervis*, *H. multiflora*), or spread across the surface of the capitulum (*E. speciosus*) for all open flowers. We replaced the pipette tip between each experimental addition to avoid cross‐pollination and cross‐contamination of nectar solutions.

Immediately after applying nectar treatments to a pair of replicate plants, we conducted 20‐min observations of floral visitors at each plant in random order, recording the identity of each visitor. We define a plant–pollinator interaction when a floral visitor comes into contact with the reproductive structures of the flower (Figure 1). For brevity, we refer to floral visitors as pollinators, keeping in mind that not all floral visits may result in pollination. Because a pollinator often visits many open flowers, we recorded the total number of flowers visited during an individual's foraging bout (“visits”), the duration of each foraging bout (“handling time”), and the number of distinct foraging bouts (an estimate of distinct “visitors” arriving at a plant during on observation session). We identified visitors in the field to the finest taxonomic unit possible. If species‐level identification was not possible in the field, pollinators were recorded as distinct morphospecies (e.g., Bain et al., 2022; Burkle & Irwin, 2009). We did not destructively sample pollinators to avoid influencing interactions during other observation periods. In total, each plant was treated and observed approximately four times depending on its flowering time.

### 2.4 Statistical analyses

#### 2.4.1 Do plants with greater sodium nectar concentration attract more floral visits by more species?

We asked whether visitation rate, number of unique visitors, handling time, and species richness differed between sodium‐enriched and control plants during each 20‐min observation period. Because the data for total visits and number of visitors were count‐based and overdispersed, we used generalized linear mixed models with negative binomial error distributions to examine whether there was a treatment effect from sodium‐enriched floral nectar (R package “glmmTMB”: Brooks et al., 2017). Our models included treatment, plant species, and sampling week as fixed effects with interaction terms (treatment × species, treatment × week). We included plant pair as a random effect to account for variation between plants of the same species across the meadow.

#### 2.4.2 Do pollinator species preferentially visit flowers with greater concentrations of sodium in their nectar?

To determine whether individual pollinator species preferentially forage on flowers with sodium‐enriched nectar, we compared the visitation rate of each pollinator species on plants with sodium‐enriched nectar to that of control plants. We used a generalized linear mixed‐effects model with pollinator species as a random effect and a Poisson distribution for the count‐based data.

#### 2.4.3 Does sodium‐enriched floral nectar significantly alter the composition of floral visitors?

We tested the effect of sodium‐enriched nectar on the composition of floral visits using multivariate analysis of variance with Bray–Curtis dissimilarities (PERMANOVA, “adonis” function in vegan package in R; Oksanen et al., 2019). We also used a multivariate homogeneity of group dispersions test (PERMDISP, “betadisper” function in vegan package; Oksanen et al., 2019) in combination with PERMANOVA to test whether nectar treatment causes differences in species composition (i.e., centroid location effect, sensu Anderson & Walsh, 2013) or a change in within treatment variation in species composition (i.e., dispersion effect, sensu Anderson & Walsh, 2013). We repeated this to test for effects of plant species identity on the composition and dispersion of floral visits. To visualize differences between the composition of floral visits among treatments and plant species, we used an ordination based on nonmetric multidimensional scaling (NMDS) of Bray–Curtis dissimilarity matrices.

#### 2.4.4 Do pollinators change their diet breadth in response to increased sodium concentrations in floral nectar?

We compared the dietary breadth for each pollinator species between treatments using Hurlbert's Probability of Interspecific Encounter (PIE). This measure is an intuitive indicator of interaction evenness and represents the probability of floral visitors selecting different resources when two are randomly selected from the community (Ellison & Gotelli, 2009; Gotelli & Graves, 1996; Semmler et al., 2022). PIE values close to 0 indicate minimum diet breadth for a given species (every interaction is the same), whereas values close to 1 indicate the maximum diet breadth (every interaction is different and in the same frequency). To calculate PIE of pollinator diets, we summed interaction observations for each plant species across all observation periods for each treatment, because each observation period or week had too few interactions to calculate a reliable PIE. From these summed interactions, we calculated the overall dietary breadth for each pollinator species in both treatments. We tested whether they varied between treatments using a Wilcoxon signed‐rank test.

All analyses were conducted in R [v. 4.1.2] (RStudio Team, 2021).

## 3 RESULTS

During 45 h of observation of our four plant species, we recorded 4351 pollinator visits to flowers from an estimated 548 distinct floral visitors. The most common floral visitors were *Bombus appositus* (26%), *B. flavifrons* (26%), and *B. bifarius* (25%).

### 3.1 Do plants with greater sodium nectar concentration attract more floral visits by more pollinator species?

**FIGURE 2:**
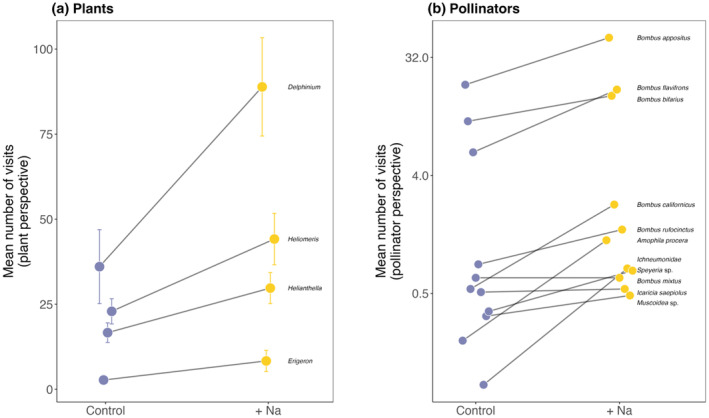
Visitation rate on control and sodium‐enriched nectar treatments for each of our four focal plant species (a) and visitation rates of *Bombus* spp. on flowers with control and sodium‐enriched nectar treatments (b). Points indicate the mean number of visits per 20‐min observation period and error bars show standard error. Visitation rates for each week by flower are shown in supplementary materials (Figure S1).

Overall, there were more than twice as many floral visits on sodium‐enriched plants per observation period (45.1 ± 6.23 SE) relative to control plants (19.8 ± 3.69 SE) throughout the season (Table 2; Figure 2a; Figure S1). The total number of visits differed among plant species (Table 2; Figure 2a), with *D. barbeyi* receiving the most (63.1 ± 9.91 SE visits), and *E. speciosus* receiving the fewest per observation period (5.53 ± 1.75 SE visits). Despite differences in overall visitation rate among plant species, the sodium‐enriched treatment effect was consistent regardless of plant species (Table 2). Visitation rates differed among weeks and increased over the season (Table 2; Figure S1). We also observed nearly twice as many distinct foraging bouts (“visitors”) on sodium‐enriched plants (5.37 ± 0.518 SE) compared to control plants (3.21 ± 0.361 SE; Table 2; Figure S2). In other words, there were more distinct visitors on plants with sodium‐enriched nectar, not just a few pollinators visiting more flowers in a single foraging bout. Overall, pollinator handling time did not vary significantly between the nectar treatments (*χ* ^2^ = 2.315, *p* = .128). Nevertheless, for *D. barbeyi*, for which we have the most data, handling time was 1.5× greater on plants with sodium‐enriched nectar (*χ* ^2^ = 9.5865, *p* = .002; Figure S4). The richness of pollinator visitors on flowers during each observation period was 35% greater on sodium‐enriched plants (1.88 ± 0.164 SE) than on control plants (1.39 ± 0.149 SE; Table 2; Figure S3).

**TABLE 2:** Summary statistics for the models describing visitation rate, visitors, and diversity. Significant effects are indicated with an asterisk (*).

Response	Fixed‐effect	*χ* ^*2*^	df	*p*
Generalized linear mixed‐effects model (family: negative binomial)				
Visitation	Treatment	14.59	1	<.001*
	Species	73.78	3	<.001*
	Week	7.78	1	.005*
	Treatment × species	0.68	3	.879
	Treatment × week	0.04	1	.835
Distinct foraging bouts	Treatment	21.67	1	<.001*
	Species	87.21	3	<.001*
	Week	9.74	1	.002*
	Treatment × species	1.06	3	.786
	Treatment × week	0.33	1	.569
Pollinator species richness (family: Poisson)	Treatment	5.133	1	.023*

### 3.2 Do pollinator species preferentially visit flowers with greater concentrations of sodium in their nectar?

Pollinator species preferentially visited flowers with sodium‐enriched nectar, on average visiting 2.7× more flowers with sodium‐enriched nectar compared to control nectar (Table 2). This pattern was consistent among all pollinator species observed in our study (Figure 2b).

### 3.3 Does sodium‐enriched floral nectar influence the composition of floral visitors?

**FIGURE 3:**
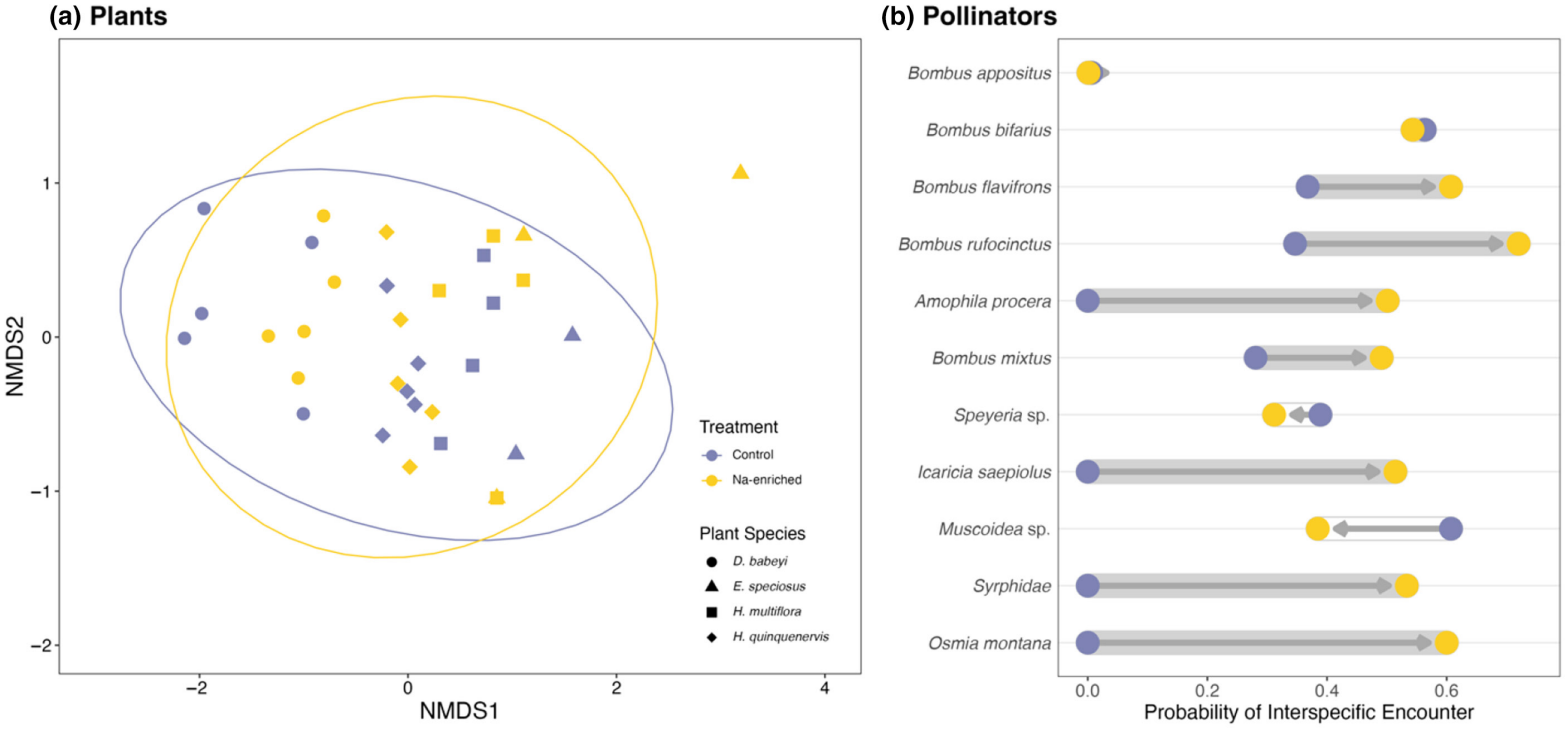
Ordination plot (NMDS) showing the community composition of visiting pollinator species for each focal plant species and experimental nectar treatment (a). Evenness of interactions for each pollinator species on flowers with sodium‐enriched (yellow) and control nectar treatments (purple) (b). Gray and white lines show an increase or decrease in Hurlbert's Probability of Interspecific Encounter, respectively. The treatment effect is further emphasized by arrows indicating the direction of change from control to sodium‐enriched treatments.

The group dispersion of the communities of pollinators visiting the plants in our experiment did not significantly differ between nectar treatments (*F* = 0.021, *p* = .88) or among plant species (*F* = 0.473, *p* = .703; Figure 3a), indicating the community composition was similar between the nectar treatments. The community composition of pollinator visitors (centroid location) not only varied mostly among plant species (*pseudo‐F* = 11.82, *p* = .001), but also nectar treatment (*pseudo‐F* = 2.17, *p* = .069; treatment × plant species: *F* = 0.99, *p* = .414). This suggests that plant species identity influences pollinator community composition more than sodium enrichment (Figure 3a).

### 3.4 How does pollinator diet breadth respond to increased sodium concentrations in floral nectar?

The presence of sodium‐enriched nectar had a significant effect on the strength of pollinator diet preference, measured as Hurlbert's Probability of Interspecific Encounter (PIE) (Gotelli & Graves, 1996; Wilcoxon signed‐rank test: *V* = 11, *p* = .027). Pollinators expanded their diets on plants with sodium‐enriched nectar, (0.47 ± 0.06 SE) compared to those with control nectar (0.23 ± 0.07 SE). Whereas most pollinators expanded their dietary breadth to include sodium‐enriched plant species (Figure 3b), there are some notable exceptions: *Bombus appositus* had nearly no change in diet breadth because it forages almost exclusively on *D. barbeyi*, and *B. bifarius* maintained a very broad diet with both nectar treatments (Figure 3b).

## 4 DISCUSSION

We found that sodium‐enriched floral nectar leads to a greater frequency and diversity of plant–pollinator interactions in a subalpine meadow. There were 2× as many floral visits and 1.5× as many pollinator species visiting sodium‐enriched plants—regardless of plant species (Figure 2a; Figure S4). These findings are consistent with observations in a different ecosystem (Finkelstein et al., 2022a) with a dramatically different set of pollinators and plant species. Our findings demonstrate that experimental nectar treatments that more closely approximate observed nectar volumes, sugar concentrations, and sodium concentrations produce a general and consistent overall effect in the context of a wild, intact plant–pollinator community. Additionally, our results were robust to temporal variation in plant–pollinator interactions in this subalpine ecosystem (CaraDonna et al., 2017)—pollinator visitation on sodium‐enriched plants was 2× greater for the 21‐day duration of the experiment (Figure S1). Taken together, our results suggest that the existing variation in floral nectar sodium concentrations (e.g., Hiebert & Calder, 1983) has the potential to shape the frequency, diversity, and composition of plant–pollinator interactions in natural communities. Nevertheless, whether plants with more sodium in their nectar experience fitness benefits remains an open question (Finkelstein et al., 2022b).

For the pollinator species observed in this experiment, we found consistent evidence that sodium is an attractive lure to flowers (Figure 2b), as it is for many other animals (Kaspari, 2020). Thus, our observation that nearly all pollinator species in the experiment appear to prefer flowers with sodium‐enriched nectar is consistent with the “salty nectar hypothesis” (Figure 1b; Kaspari, 2020). Pollinators depend heavily on nectar (and pollen) for their nutrient demands, and most pollinators lack strategies to supplement sodium (*but see*: Bänziger et al., 2009, Dorian & Bonoan, 2021), which might have been a significant hurdle in the evolution of pollen‐ and nectar‐eating bees (Filipiak et al., 2023). The importance of sodium intake is likely linked to maintaining digestive, excretory, and neuromuscular systems (Molleman, 2010). We observed that not only did pollinators visit more flowers, they increased their interaction evenness on sodium‐enriched plants (Figure 3b). That is, nearly all pollinator species expanded their diet breadth to maximize the sodium reward (with few exceptions: *Bombus appositus* showed almost no change because it foraged almost exclusively from one plant species, *D. barbeyi*, and *B. bifarius* remained a generalist and visited all four plant species relatively evenly regardless of treatment).

Sodium in floral nectar may also affect other aspects of pollinator foraging behavior. For instance, some pollinators may spend more time on plants with sodium‐enriched floral nectar (Figure S3). Evidence from *D. barbeyi* suggests that among *Bombus* spp. (a group with relatively generalized foraging habits and the most frequent pollinator in our study; Ogilvie & CaraDonna, 2022), handling time was about 1.5× greater on flowers with sodium‐enriched nectar. But we did not observe similar evidence for the other plant species, *Helianthella quinquenervis*, for which we had sufficient handling time data (Figure S3). This inconsistency may be driven by considerable differences in flower morphology of the two species: *D. barbeyi* has flowers with long nectar spurs and copious nectar and *H. quinquenervis* has numerous small flowers arranged as a capitulum, each with a small volume of nectar. These patterns suggest that pollinator foraging behavior may be affected by micronutrient availability, but as of yet, it is unclear how or if this variation in handling time could influence the quality of pollination services (Ivey et al., 2003; Murúa, 2020).

Not only did we find that flowers with sodium‐enriched nectar attracted more visits, we also observed that flowers with sodium‐enriched nectar attract a greater number of distinct visitors (Figure S2) as well as a greater richness of pollinators (Figure S3). Together, these findings suggest that pollinators can detect sodium‐enriched floral nectar at a distance. Yet, it currently remains unclear how pollinators might recognize and detect sodium‐enriched nectar. Pollinators can perceive nectar volatiles with their antennae and increase visitation on high reward flowers compared to flowers with depleted nectar (e.g., Howell & Alarcón, 2007). Although sodium does not volatilize (Pauling, 1988), salt in nectar can modify volatile organic compounds and even increase the potency of scents through the process of “salting‐out,” whereby salts increase the solubility of neutral substances in water (Sergeeva, 1965). Additionally, it is possible that sodium alters nectar yeast communities and their scents (Klaps et al., 2020; Mostafa et al., 2022; Schaeffer et al., 2017). In both cases, such sodium‐induced changes to volatiles within floral nectar would attract more pollinators to the flowers. Alternatively, pollinators may have recognized, learned, and remembered the sodium‐enriched nectar like other high‐quality floral rewards (Dukas, 2008; Dukas & Visscher, 1994; Papaj & Prokopy, 1989; Wright et al., 2013). However, in the context of our field experiment, this would require individual pollinators to regularly return to the same plant more than once within a single 20‐min observation session or remember over several days that only some plants in the meadow—and only from time to time—have sodium‐enriched nectar. Others have argued that optimal foraging may explain the increase in pollinator visitation, with visitors finding a plant especially rewarding and visiting more flowers on plants with sodium‐enriched nectar (Pyke & Ren, 2022). This is plausible, but it does not on its own explain the increase in the number of distinct visitors (Figure S3) and the increase in the richness of pollinators on sodium‐enriched plants (Figure S4), which at a minimum suggests pollinators must be able to detect sodium‐enriched nectar (Finkelstein et al., 2022b).

Changes to the frequency and diversity of floral visitors were accompanied by a shift in the community composition of visitors on flowers with sodium‐enriched nectar. Sodium enrichment of floral nectar altered the community composition of floral visitors, even though the plant species effect was most prominent and temporal variation obscured some treatment effects (Figure 3a). Thus, temporal and geographic variation in the availability of sodium may contribute to turnover and rewiring of plant–pollinator interactions (CaraDonna et al., 2017). However, it remains unclear whether the increase in sodium availability tangibly changes the structure, resilience, and effectiveness of the entire network. To better understand the role of sodium in nectar at the community and network‐level, future studies that simultaneously manipulate nectar properties in multiple plants in a plot could observe how a manipulated community, rather than individual plants, affects the structure and function of plant–pollinator interaction networks.

Presumably, the increase in floral visitors, richness, handling time, and diet breadth may confer a fitness advantage for sodium‐enriched plants, which can then drive the evolution of sodium in nectar. However, few studies have definitively linked pollinator preference of nectar traits to changes in plant fitness (Parachnowitsch et al., 2019). Longer handling times on plants with more nectar have been shown to increase seed set (Brandenburg et al., 2012; Jennersten, 1988; Manetas & Petropoulou, 2000), but other evidence suggests that this variation can have little effect on plant reproduction (Adler & Irwin, 2005). What is needed is a link in pollinator response to nectar traits—like sodium concentrations in nectar—to seed set and reproduction (e.g., Mitchell, 1994) to determine whether the variation in sodium concentrations (Hiebert & Calder, 1983) can affect fitness and demography. Furthermore, it is unclear how much variation in nectar traits—not only sodium concentrations—occurs because of environmental variation (Enkegaard & Boelt, 2016; Waser & Price, 2016) versus heritable variation (Mitchell, 2004; Parachnowitsch et al., 2019). This gap highlights a need for research to address our limited understanding of the variation of nectar sodium within and across plant communities and species—especially given the broad importance of sodium limitation for many animals. Addressing these areas will have implications as sodium availability shifts because of altered ungulate distributions and their urine inputs (McNaughton et al., 1997), runoff from road salts (Mattson & Godfrey, 1994), and broad‐scale patterns from changing precipitation (Hassani et al., 2021). Especially in sodium‐poor regions far from the coast (e.g., Kaspari, 2020; Kaspari et al., 2014), changes in sodium availability may alter plant physiology, the abundance of invertebrate consumers (Filipiak et al., 2023; Kaspari & Welti, 2023), and ultimately the frequency and structure of plant–pollinator interactions.

## AUTHOR CONTRIBUTIONS

**Ethan VanValkenburg:** Conceptualization (equal); data curation (lead); formal analysis (lead); investigation (lead); methodology (equal); visualization (lead); writing – original draft (lead); writing – review and editing (equal). **Thiago Gonçalves Souza:** Formal analysis (supporting); writing – review and editing (equal). **Nathan J. Sanders:** Conceptualization (equal); formal analysis (supporting); supervision (equal); writing – review and editing (equal). **Paul CaraDonna:** Conceptualization (equal); formal analysis (supporting); funding acquisition (lead); investigation (supporting); methodology (equal); resources (lead); supervision (equal); visualization (supporting); writing – review and editing (equal).

## FUNDING INFORMATION

PJC was supported by NSF Grant DEB‐1754518; ideas for this project were spawned in NSF Grant DEB‐1556185 to NJS.

## CONFLICT OF INTEREST STATEMENT

The authors declare no conflict of interest.

## Supporting information


Figures S1–S4.

## Data Availability

All data are available at DOI: 10.5061/dryad.7h44j1018. All R scripts for analysis and figures are available on GitHub (https://github.com/vanvalke/salty‐nectar.git).
