# Supplementary material for: Sodium‐enriched nectar shapes plant–pollinator interactions in a subalpine meadow
Source: Ecol Evol. 2024 Jul 16;14(7):e70026. doi: 10.1002/ece3.70026 (PMC11251754; doi:10.1002/ece3.70026)
Supplement: Supplementary file 1 — Figures S1–S4. [file ECE3-14-e70026-s001.docx]

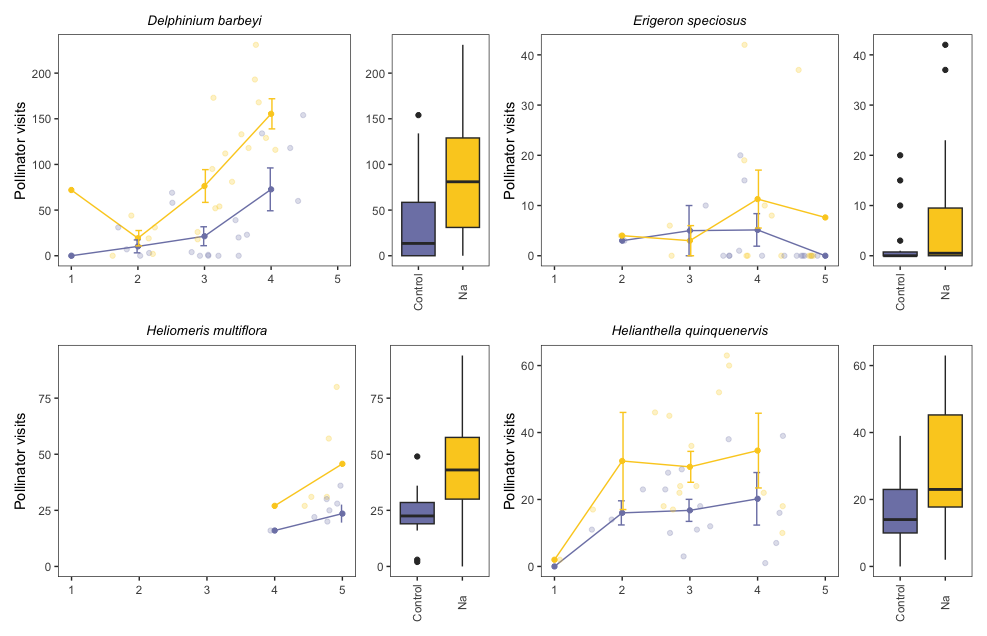


**Figure S1.** Pollinator visitation with control and sodium-enriched nectar treatments for all 4 plant species by sampling week. Error bars indicate standard error and dots represent the number of pollinator visits on a plant during a single 20 min. observation period. Right margin boxplots show visitation rate across the entire season. The frequency of pollinator visits is always greater on sodium-enriched plants compared to the control, regardless of plant species or week.


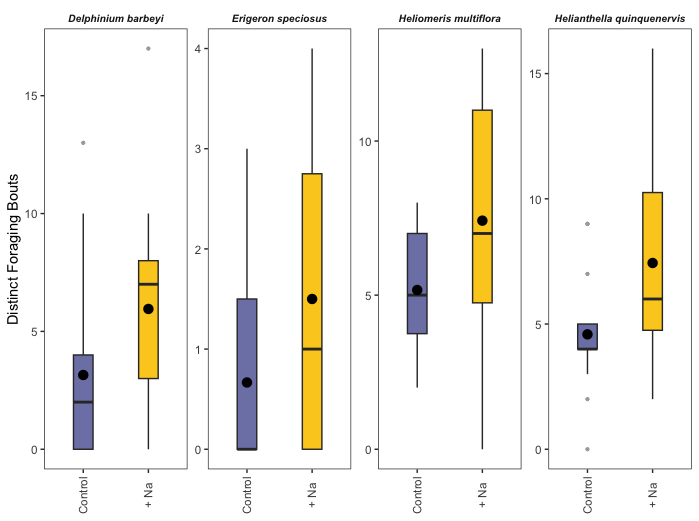


**Figure S2.** The effect of sodium enrichment on the number of distinct foraging bouts (ie. unique visitors) on each plant species. Points indicate the mean number of visitors. The number of unique visitors is always greater on sodium-enriched plants compared to control plants, regardless of plant species.


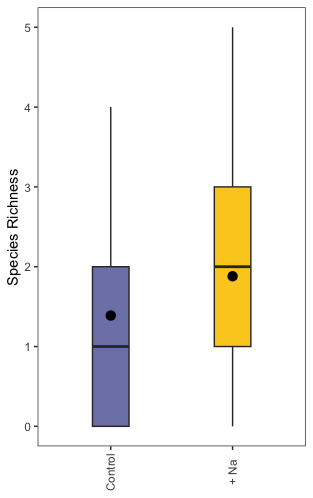


**Figure S3.** Species richness of visitors on sodium-enriched compared to control plants. The diversity metric was calculated for each 20-minute observation period on each flower. Points indicate the mean richness.

# **
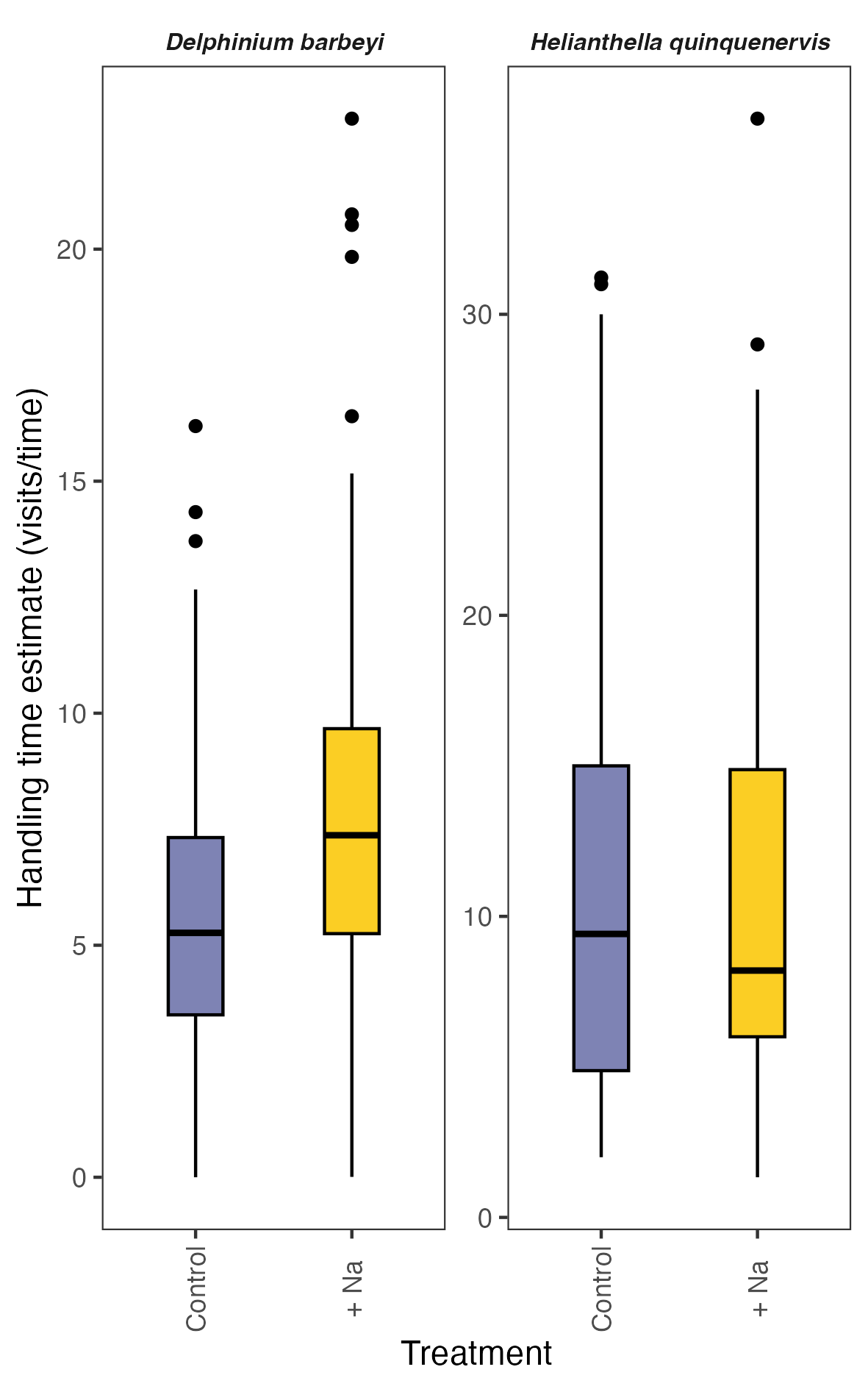
**

**Figure S4.** Handling time with sodium-enrichment. We estimated the handling time using the most abundant pollinator group with sufficient replication (n = 176): bumble bees (*Bombus* spp). We calculated handling time by dividing the total duration (in seconds) of each foraging bout by the number of flowers visited by an individual pollinator. The linear mixed-effects model included handling time and plant species as response variables. We included treatment, week, and plant species as fixed effects with interactions terms and pair number as random effects, as with our other models. Although *Bombus* spp. visited all plant species, we only have sufficient replication to include *D. barbeyi* and *H. quinquenervis* in this model. Our results show that handling time of *Bombus* visitors did not vary significantly with the sodium-enriched treatment (*χ²* = 2.315, *p* = 0.128). Plant species, however, did have a significant effect (*χ²* = 35.8736, *p* < 0.001). For only *Delphinium barbeyi* visitors*,* handling time was approximately 1.5× longer (*χ²* = 9.5865, *p* = 0.002) in the sodium-enriched treatment compared to the control. The different responses between plant species may be driven by the difference in flower morphology of the two species: namely *D. barbeyi* has flowers with long nectar spurs and *E. speciosus* has flowers as a capitulum
